# Large-Scale Evaluation of Collision Cross Sections to Investigate Blood-Brain Barrier Permeation of Drugs

**DOI:** 10.3390/pharmaceutics13122141

**Published:** 2021-12-13

**Authors:** Armin Sebastian Guntner, Thomas Bögl, Franz Mlynek, Wolfgang Buchberger

**Affiliations:** Institute of Analytical and General Chemistry, Johannes Kepler University, 4040 Linz, Austria; thomas.boegl@jku.at (T.B.); franz.mlynek@jku.at (F.M.); wolfgang.buchberger@jku.at (W.B.)

**Keywords:** collision cross section, drug, blood-brain barrier, cerebral pharmacokinetics, molecular descriptor

## Abstract

Successful drug administration to the central nervous system requires accurate adjustment of the drugs’ molecular properties. Therefore, structure-derived descriptors of potential brain therapeutic agents are essential for an early evaluation of pharmacokinetics during drug development. The collision cross section (CCS) of molecules was recently introduced as a novel measurable parameter to describe blood-brain barrier (BBB) permeation. This descriptor combines molecular information about mass, structure, volume, branching and flexibility. As these chemical properties are known to influence cerebral pharmacokinetics, CCS determination of new drug candidates may provide important additional spatial information to support existing models of BBB penetration of drugs. Besides measuring CCS, calculation is also possible; but however, the reliability of computed CCS values for an evaluation of BBB permeation has not yet been fully investigated. In this work, prediction tools based on machine learning were used to compute CCS values of a large number of compounds listed in drug libraries as negative or positive with respect to brain penetration (BBB^+^ and BBB^−^ compounds). Statistical evaluation of computed CCS and several other descriptors could prove the high value of CCS. Further, CCS-deduced maximum molecular size of BBB^+^ drugs matched the dimensions of BBB pores. A threshold for transcellular penetration and possible permeation through pore-like openings of cellular tight-junctions is suggested. In sum, CCS evaluation with modern in silico tools shows high potential for its use in the drug development process.

## 1. Introduction

**Figure 1:**
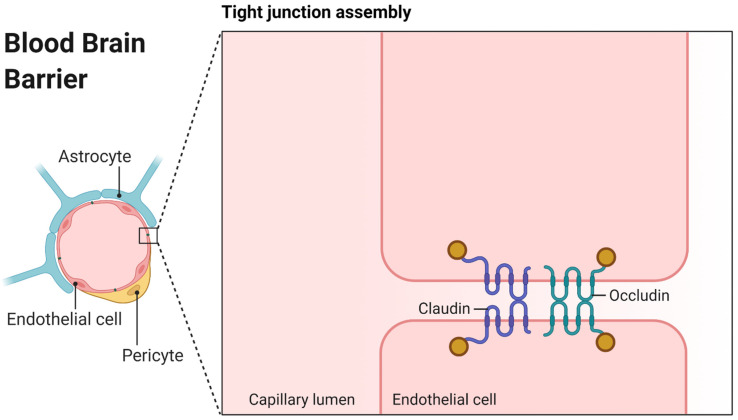
Graphical representation of the blood-brain barrier (BBB) created with BioRender.com (accessed on 8 November 2021). The left panel shows the important cellular parts, while the right zoomed panel shows the tight junction assembly in detail.

Successful brain therapy places high demands on the molecular properties of drugs. Besides activity towards cerebral target sites, appropriate cerebral pharmacokinetics (PK) are also essential. Unfortunately, most drugs do not sufficiently reach the brain [1,2]. A primary obstacle drugs must overcome to enter the central nervous system (CNS) is the blood-brain barrier (BBB), a complex protective shield, built up first of all of endothelial cells at the epithelium of the vascular network of the brain [3,4]. Here, tight junctions employing proteins such as occludin or the claudin superfamily seal the intercellular crevice (see Figure 1) [5]. On the cellular level, the neurovascular unit is composed of endothelial cells, a basal lamina, pericytes and astrocytes, the latter of which are relevant in inducing and maintaining the barrier function and linking to neurons [6].

Therefore, drugs mainly enter the brain mainly transcellularly across the endothelial cells [6,7]. To avoid exposure of the brain exposition to undesirable substances, these cells express efflux pumps that exfiltrate, e.g., xenobiotics under energy consumption. If substances are substrates to efflux systems such as the permeability glycoprotein (P-gp), brain exposure is significantly reduced. On the other hand, active uptake routes are also present, assuring proper supply of the brain with nutrients [6,7].

Besides the mentioned active transportation, passive diffusion across the blood-brain barrier (or in more detail across the lipid-double layer of the endothelial cells) may occur. The direction of the diffusion obeys the concentration gradients present [6]. Whether a drug will actually arrive at the CNS is thereby dependent on numerous structure-derived parameters including lipophilicity, molecular weight, the number of hydrogen bond donors and acceptors, and the polar surface area (PSA) as well as molecular volume, flexibility, and size (in membrane-bound conformation) [8,9,10,11,12].

A recent study by our group showed that collision cross sections (CCS) can be used in this field, as they are characteristic for the molecular structure (reflecting branching, flexibility and molecular volume among other aspects) and correlate significantly with molecular mass [13,14]. Spherical molecules appear generally superior to elongated or bulky structures in terms of cerebral PK properties [9]. In fact, one will intuitively assume that a compact molecule can penetrate a membrane layer more easily than a bulky one. This characteristic is perfectly reflected in the rotationally averaged collision cross section, as shown in the graphical abstract.

For the experimental determination of CCS, drift-time ion mobility instruments can be used that measure the time that ions (generated by initial ionization) need to travel a defined distance. Here, compact ions pass a drift tube filled with a buffer gas such as nitrogen faster than elongated ions of the same mass. In other words they exhibit a higher ion mobility in the gas phase [14,15]. The mobility of an ion is dependent on the mass and the charge of the ions, but is also significantly dominated by its shape and size. Mathematically this relation is represented by the Mason–Schamp equation (Formula (1)) that correlates CCS with ion and buffer gas mass, arrival time, and a few other parameters [15,16,17]:

(1) CDTCSN2=18π16zekbT1mi+1mBtAEL760PT273.151N

z and e charge number and elementary charge, $k_{b}$ Boltzmann constant, T drift cell temperature, $m_{i}$ and $m_{B}$ ion and buffer gas mass, $t_{A}$ measured arrival time, E applied electrical field, L drift tube length, P pressure in the drift cell and N neutral gas number density [15].

CCS values are in fact gaining importance in the characterization of drugs [18]. This parameter may also be calculated in silico on the basis of the molecular structure [15,19]. The advantage thereby is that no pure drug compound is necessary and the computation is fast. Here, the accuracy of computed CCS does not only depend on the performance of the calculation tool but also on the quality of the input data. Accordingly, input data based on a low-level of theory allow only approximate results with limited reliability [13]. Fortunately, recent developments have overcome the dependency on the quality of the input data because modern high-performance prediction tools solely rely on the unambiguous Simplified Molecular Input Line Entry Specification (SMILES). In this field, two efficient options should be considered that are freely available online: AllCCS from ZhuLab [20] and CCSbase from Libin Xu Lab [21]. Both allow highly accurate and batchwise calculation of CCS [20,21], so that large-scale computation of this descriptor becomes feasible.

Setting general CCS limits for sufficient BBB penetration of drugs expands the list of available descriptors and supports pharmaceutical development. Further, CCS includes spatial information that can be used to further characterize the permeation of drugs in the biological system, meaning the passive diffusion across the blood-brain barrier. In this work, four drug libraries including substances of known and predicted CNS activity were correlated with computed CCS, and the prediction performance of calculated CCS was evaluated. One dataset of more than 1500 substances was evaluated with Random Forest analysis to compare CCS with other molecular descriptors in terms of its ability to differentiate substances with and without reported CNS activity. This library was initially compiled by Adenot and Lahana [22] but was improved considerably by Zhao et al. who added molecular properties to the listed substances [23]. In this compilation, substances denoted as CNS^+^ or BBB^+^ respectively, are known to exhibit CNS activity, in other words trigger effects at CNS target sites. CNS^−^ or BBB^−^ drugs are substances, which lack such activity. This may be attributed to insufficient BBB penetration or to penetration without apparent CNS efficacy due to a lack of matching target sites [22]. CCS were also computed for all suitable substances listed in the Drugbank [24], as a comprehensive correlation of computed CCS and library data may promote the understanding of BBB penetration. Another library of 400 compounds (compiled by Li et al. [25]) was considered for prediction performance tests of CCS in direct comparison with the BOILED-Egg approach [26] that is used by the SwissADME webserver [27]. All mentioned data sets and a fourth library of more than 350 compounds curated by Muehlbacher et al. [28] were used to test single descriptor prediction accuracy of CCS. The corresponding data of all investigated substances and related descriptors are given in the Supplementary Material.

## 2. Materials and Methods

### 2.1. Statistical Evaluation of CCS as a Molecular Descriptor of BBB Penetration

Initially the performance of CCS for the prediction of BBB penetration of compounds was evaluated. For this purpose a dataset used by Adenot and Lahana [22] and curated by Zhao et al. [23] was considered. This set was derived from the comprehensive world drug index database and Adenot and Lahana filtered substances with denoted CNS activity. Zhao et al. then cleaned and extended the dataset and listed BBB^+^ and BBB^−^ compounds. Of all listed substances in their work, 1592 compounds of known BBB permeability (1282 BBB^+^, 310 BBB^−^, ratio 4.1) were then included in our investigations. BBB permeability given in literature was denoted according to actual drug activity in the central nervous system. The listed SMILES codes of Zhao et al. [23] were used to compute CCS values of (M+H)^+^ and (M-H)^−^ adducts of the individual compounds using CCSbase.net web interface [21]. According to the batch prediction instructions given on this website (https://ccsbase.net/predictions, last time accessed on 8 December 2021), following the example .csv file), SMILES codes of the substances were uploaded as a .csv file and predictions were conducted.

CCSbase.net is a machine-learning-based in silico tool that allows batchwise prediction of CCS with ease while assuring high accuracy (>90% of the test set within 5% difference to measured values) [21]. According to the predominant charge (positive or negative) at physiological pH as given by Zhao et al. [23] either (M+H)^+^ or (M-H)^−^ adducts were calculated and considered. In the case of neutrals (M+H)^+^ adducts were used. This approach was chosen to mimic the biological scenario more accurately than previous approaches, where adduct selection was predetermined by chemical properties and electrospray ionization respectively [13].

Secondly a large compound set based on the Drugbank [24] was investigated. After initial data cleansing more than 3000 compounds (2841 BBB^+^, 420 BBB^−^, ratio 6.8) were evaluated. In detail, all accessible Drugbank data were screened for available SMILES code to predict CCS with the CCSbase.net web interface as well as for computed BBB permeation data with a prediction probability > 0.9. Importantly, Drugbank provides solely predicted BBB values on the basis of admetSAR computation [29].

In both cases, statistical evaluation was performed with Metaboanalyst [30] to test for parameter importance for group differentiation (BBB^+^/BBB^−^). For that purpose, the data was uploaded and grouped according to BBB penetration properties. Random Forest analysis was employed without normalization, scaling or data filtering to test for variable importance and prediction accuracy. Randomness was turned off and the constant 123,456 was used. Eight molecular descriptors were initially considered, but were subsequently decreased stepwise to six and four. All tested features were numerical; therefore, no additional data processing steps were necessary. Random Forest analysis was performed with 1000 trees in each case. Detailed information on the working principle of Random Forest analysis is given in [31].

To test the investigated datasets for scaffold diversity we chose the approaches presented by Langdon et al. and Chhabra et al. [32,33]. In this manner, we used DataWarrior Chemical Data Analysis and Visualization software (v5.5, openmolecules.org) to analyse scaffold diversity with its built-in application. The results of the analysis are shown in Table 1. All datasets exhibit high scaffold diversity and expand over the whole chemical space according to the high relative number of scaffolds compared to the number of compounds tested.

### 2.2. Performance Evaluation of CCS Next to the BOILED-Egg Approach

Another analysis was conducted to test the actual prediction performance of CCS for blood brain-barrier penetration in comparison with the BOILED-Egg approach [26]. For this purpose, a data collection of 400 compounds (267 BBB^+^, 133 BBB^−^, ratio 2.0) originally published by Li et al. [25] and curated by Zhao et al. [23] was tested. Substances were grouped in the literature as crossing/not crossing the BBB according to the logarithmic ratio of drug concentration in brain and blood (log BB; BBB^+^ log BB ≥ −1, BBB^−^ log BB < −1) [23,25]. Available SMILES codes were used to compute molecular weight, polar surface area, and lipophilicity using SwissADME webserver [27]. In addition, the available SMILES codes were used to predict CCS of (M+H)^+^ adducts with CCSbase.net web interface [21]. In the next step compounds were assigned to the BBB^+^ group if XlogP3 > −1 and/or CCS < 200 Å^2^ according to limits given in the literature for these descriptors of BBB permeation [13,34]. Finally CCS prediction performance was compared with the performance of the SwissADME webserver, that relies on the previously mentioned BOILED-Egg approach considering PSA and lipophilicity [26]. The results of both approaches were compared with the literature data on BBB permeation of drugs for accuracy evaluation.

### 2.3. Performance Evaluation of CCS as a Single Molecular Descriptor

Finally, all mentioned sets originating from Adenot et al., Li et al. and the Drugbank [22,24,25] as well as another library curated by Muehlbacher et al. [28] were used to test the prediction accuracy using only CCS values as a single descriptor. The set of Muehlbacher (327 BBB^+^, 35 BBB^−^, ratio 9.3) was based on experimental data and substances were again grouped according to the listed logarithmic ratio of drug concentration in brain and blood (log BB; BBB^+^ log BB ≥ −1, BBB^−^ log BB < −1). Also in this case, the available SMILES codes were used to predict CCS of (M+H)^+^ adducts with CCSbase.net web interface [21]. If the calculated CCS appeared to be above 200 Å^2^ compounds were predicted as BBB^−^. The detailed workflow is given in the Results and Discussion section. In all cases, the results of the CCS-based prediction were compared to literature data, namely admetSAR computation in the case of the Drugbank for performance evaluation.

The software packages included in this study were MS Excel, ccsbase.net interface, Metaboanalyst 5.0, SwissADME webserver, and DataWarrior software.

## 3. Results and Discussion

**Figure 2:**
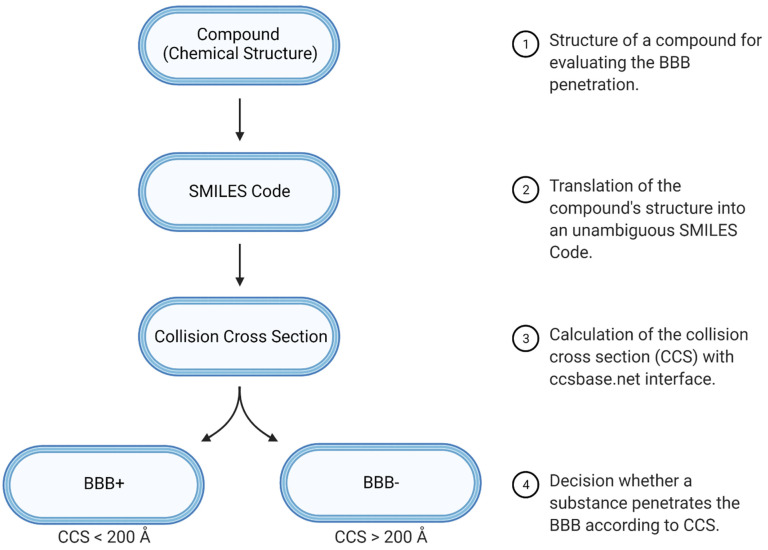
Workflow to predict CCS for evaluating BBB penetration properties of drugs. Created with BioRender.com (accessed on 8 November 2021).

The implementation of CCS in a predictive workflow for a comprehensive understanding of cerebral pharmacokinetics of a drug is fast and easy and can be achieved with open access tools that are available online. This is a promising option already early in the drug development process for optimizing lead compounds in all further steps of this process. As is shown in this work, the unambiguous SMILES code of a molecule allows the calculation of its CCS and a subsequent prediction of possible brain penetration. The high value of this prediction can be attributed to a few major aspects of this study that are displayed in detail below. The design of the study with regard to the workflow for predicting BBB penetration is given in Figure 2.

### 3.1. Random Forest Data Evaluation

**Figure 3:**
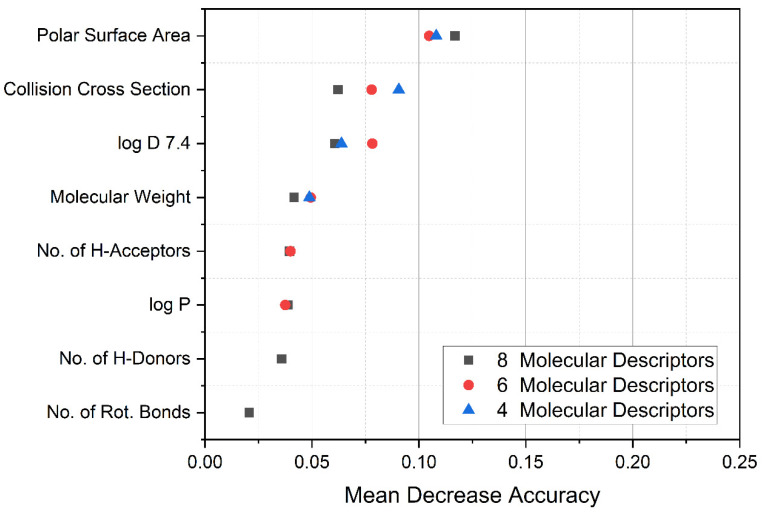
Results of Random Forest evaluation using Metaboanalyst to test variable importance for group differentiation (BBB^+^ or BBB^−^ compound) in a set initially compiled by Adenot et al. [22]. A stepwise reduction of parameters employing 1000 trees shows the dominant molecular descriptors. Mean Decrease Accuracy describes the reduction of the prediction performance of a theoretical model when a parameter is omitted and is a measure of parameter importance.

First, a Random Forest data evaluation of 1592 compounds of known BBB permeability (BBB^+^ and BBB^−^) reveals the most important descriptors for group discrimination (see Figure 3). Mean decrease accuracy in this case describes the loss of the model’s accuracy if a parameter is omitted and characterizes therewith descriptor importance. In agreement with the literature [35], the polar surface area is the predominant molecular descriptor in the present case. This descriptor is followed by the collision cross section and the lipophilicity at physiological pH. Although the CCS is mass-dependent, the information content of the CCS is obviously not limited to the mere molecular mass, but additionally includes molecular volume, structure, and flexibility, which explains the increased importance. Indeed, these mentioned aspects are known to have an impact on the penetration of drugs into the brain. In other words, spherical molecules penetrate better than stretched or bulky ones [9].

Importantly, for four molecular descriptors, the out-of-bag error (relative amount of false predictions) was determined to be as low as 6.1% with class errors of BBB^+^ 2.5% and BBB^−^ 21.3%. The higher class-error of BBB^−^ is most probably attributed to the composition of the dataset as substances that may penetrate the BBB but lack CNS activity are categorized as BBB^−^ here.

In addition, different model accuracies have been achieved by utilizing Jupyter-Lab 3.2.4 and Python 3.9. The following libraries were used for data preparation, model training and validation, and visualization: numpy 1.19.5, pandas, matplotlib 3.4.3, seaborn 0.11.2, scipy 1.7.1 and scikit-learn 1.0.1. The evaluation was performed on the Adenot et al. dataset. For the training-test split a test size of 0.3 and a random state of 42 was chosen. K-fold cross validation was done and set to 100. Further model specific parameters can be retrieved from the additionally provided jupyter notebook BBB_DataAnalysis.ipynb and the results are given in the Supplementary Material file.

### 3.2. Visualization of Compound Properties for BBB Penetration

**Figure 4:**
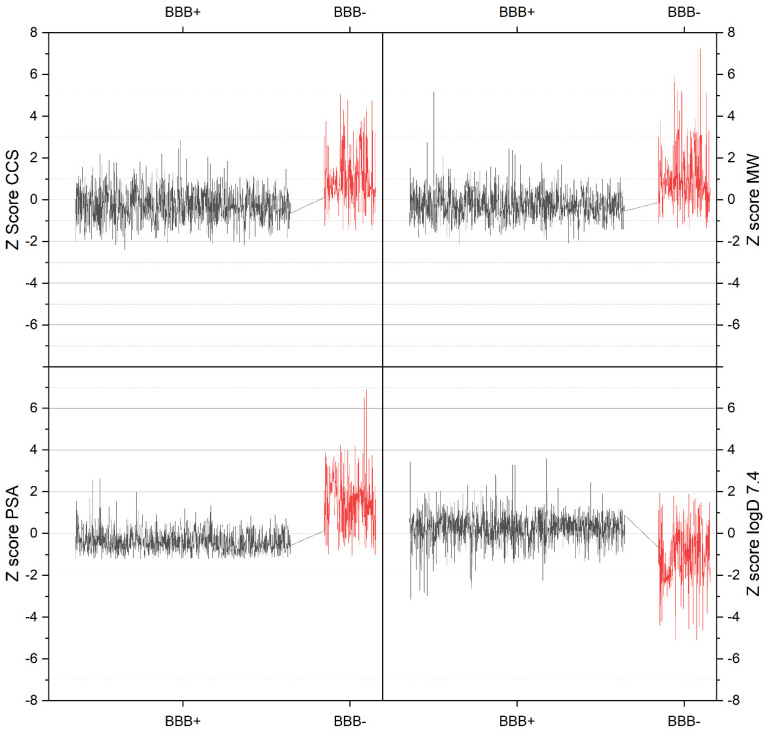
Depiction of Z score normalized individual values of collision cross section (CCS), molecular mass, polar surface area, lipophilicity at physiological pH (log D 7.4) for substances with and without CNS activity respectively BBB penetration (denoted as BBB^+^ and BBB^−^).

In a next step, Z score normalization was used to depict the data according to collision cross section, molecular weight, polar surface area and lipophilicity at physiological pH as to be seen in Figure 4. The Z score uses the difference between raw score and mean of the population divided by the standard deviation of the population for normalization. Here a clear difference between BBB^+^ and BBB^−^ compounds is visible for every molecular descriptor. A comparison of compounds with and without BBB penetration shows that smaller CCS values agree well with better penetration. This is also true for polar surface area and molecular mass. In contrast, lower lipophilicity reduces the probability of CNS activity. These aspects agree well with common predictions [8] and provide tendencies that should be taken into account in developing new CNS drugs. In fact, BBB^+^ compounds should exhibit decreased CCS, molecular mass, and polar surface area and increased lipophilicity when solely passive diffusion is considered. It is possible that substrate interaction with transporters may fully change the overall behavior of a compound, but was not considered in this work that focused solely on passive diffusion behavior.

**Figure 5:**
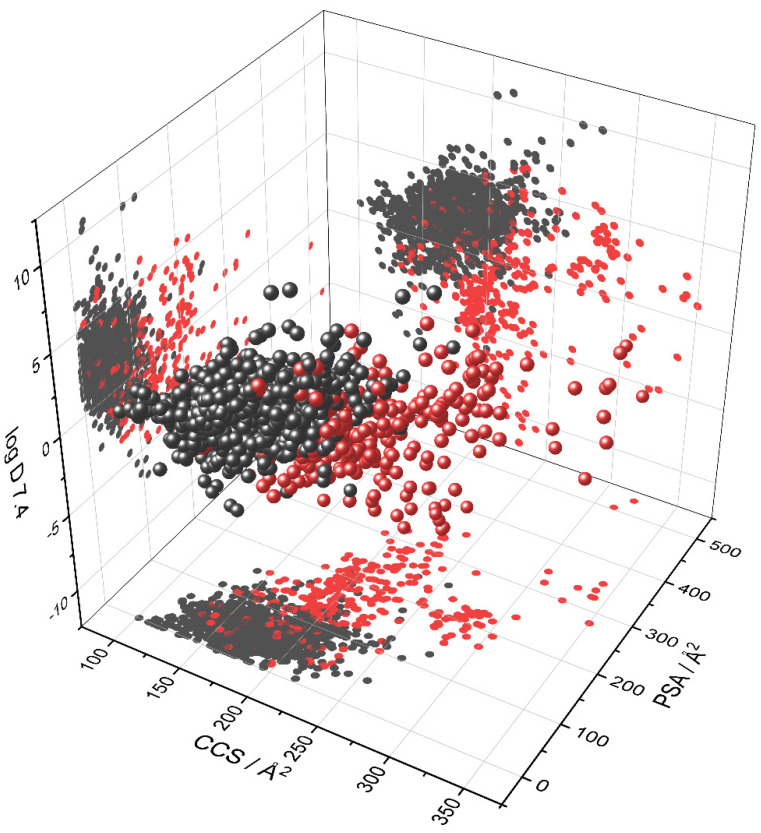
Depiction of compounds initially used by Adenot et al. [22] according to lipophilicity, collision cross section and polar surface area. Red color indicates BBB^−^ compounds and black BBB^+^ respectively.

For better comprehensibility, Figure 5 shows the correlation of the three predominant molecular descriptors (that were identified with Random Forest data evaluation as shown in Figure 3) and BBB penetration in a three-dimensional plot. A distinctive clustering is visible in the three-dimensional presentation and the related two-dimensional projections. As is obvious, none of the descriptors alone is capable of group discrimination, which is one major problem to the field. In other words, no single parameter alone allows differentiation for all possible pharmaceutically active compounds. In fact, most of the time a combination of multiple parameters is needed for a meaningful prediction as to whether a substance will enter the central nervous system or not. For the sake of completeness, Table 2 provides general limits for multiple molecular descriptors in the case of successful CNS drugs.

### 3.3. Drugbank Evaluation Regarding the BBB

**Figure 6:**
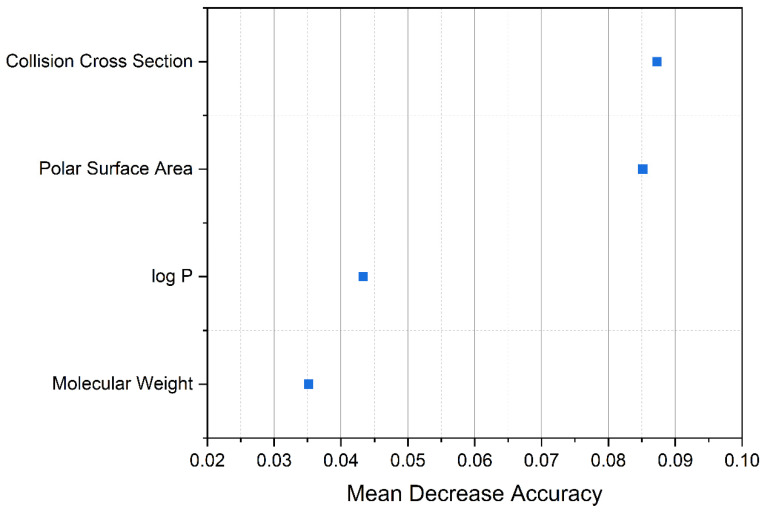
Random Forest evaluation of 4 descriptors and 1000 trees applied to the Drugbank data set. Mean Decrease Accuracy describes the reduction of the prediction performance (BBB^+^ or BBB^−^) of a theoretical model when a parameter is omitted.

**Figure 7:**
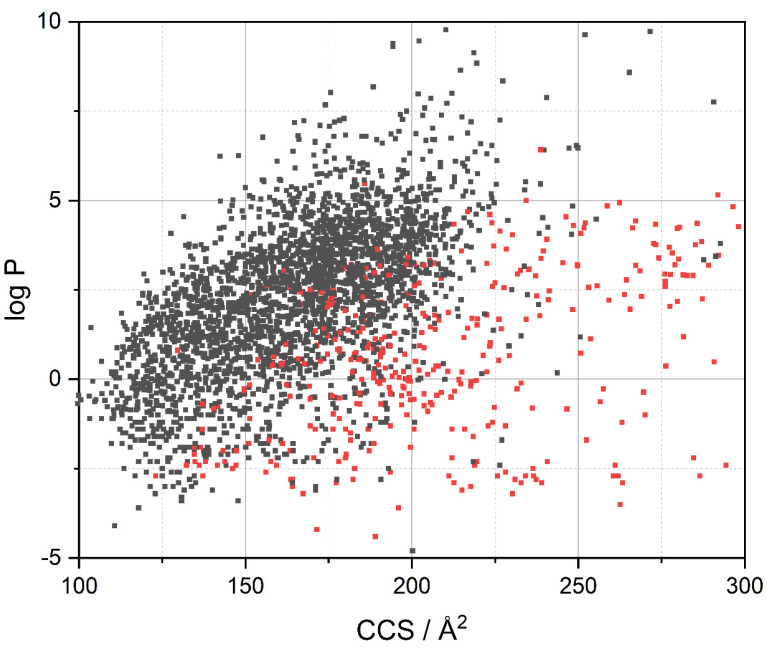
Visualization of the Drugbank library according to lipophilicity and collision cross section. Red color indicates BBB^−^ compounds and black BBB^+^ respectively.

An evaluation of the Drugbank data as another set could prove the high value of CCS for predicting BBB permeation of drugs. Random Forest analysis of 4 descriptors and 1000 trees shows that CCS and PSA are virtually similar important descriptors for group differentiation. The corresponding information is displayed in Figure 6. In addition, for better visibility of the clustering induced by CCS, Drugbank data was plotted in Figure 7. The assignment of BBB^−^/BBB^+^ was done according to admetSAR [29] prediction and represents therefore theoretical rather than empirical grouping.

### 3.4. Prediction Performance of CCS for Evaluating BBB Penetration Properties

In a next step, a comparison of the prediction performance of CCS combined with lipophilicity and the performance of the BOILED-Egg approach [26] was conducted. Considering the data set of Li et al. excluding substances with erroneous SMILES code, a total of 400 compounds (BBB^+^ 267, BBB^−^ 133) were investigated. The prediction accuracy was evaluated in comparison to literature data. While the BOILED-Egg approach that uses lipophilicity and PSA [26] resulted in an accuracy of 67% according to SwissADME output, our CCS/XlogP3 approach showed a prediction accuracy of 71%. This performance looks promising and it must be noted that no information on the physiologically present charge was available here, which is why only (M+H)^+^ adducts were used. A consideration of the physiological charge would even further increase the prediction accuracy, which has been shown by preliminary tests on the Adenot set [22].

The prediction performance for BBB penetration using only CCS was finally tested with sets of Adenot et al., Li et al., Muehlbacher et al. and the Drugbank [22,23,24,25,28]. For that purpose, CCS values that were computed on the basis of listed SMILES code (given in the Supplementary Material) were used to predict BBB penetration as shown in the workflow scheme (Figure 2). Substances were denoted as BBB^+^ if the CCS was below 200 Å^2^. The following prediction accuracies could be achieved: Adenot set 80%, Li set 66%, Muehlbacher set 86% and Drugbank set 86%. These prediction accuracies are high, especially considering the fact that a prediction based on multiple descriptors may only allow for a similar performance [28]. The differences in the prediction performance may be attributed to the composition of the sets but also to the different methods of classification used by the authors of the sets. While the Adenot set is based on actual cerebral effects, the Li and Muehlbacher sets are described with respect to log BB and the grouping of the Drugbank data are based on admetSAR predictions. Li et al. and Muehlbacher et al. both refer to log BB but reported different grouping borders. We went for the borders of Li et al. in both cases for conformity.

### 3.5. Correlation of CCS with Pore Dimensions in the Brain

A final aspect that needs to be addressed is the comparison of numerical CCS and dimensions of pores present within the blood-brain barrier in the biological system. The CCS is essentially the projection of a sphere formed by a freely rotating molecule in the gas-phase taking into account the nature of the buffer gas (nitrogen in most cases). In detail, the CCS is defined as the resulting area considering the radii of both colliding objects (neutral buffer gas and investigated ion/molecule). However, considering both objects as hard spheres tends to ignore the fact that in reality a measured CCS is more a momentum transfer cross section rather than a real collision cross section. By convention both labels may be used synonymously [36] but it should be noted that the momentum and collision cross section may differ by up to 40% [37].

In any case, it is possible to transform the calculated or measured CCS into radii of molecular spheres. For the Adenot set [22] set it could be deduced that for BBB^+^ compounds the mean CCS is 174 Å^2^ (±25 Å^2^ SD). For the transformation of the mean CCS into projected ion areas and the related radii, the following consideration is important. In a hard sphere model, a collision between compounds (that form spheres upon rotation in all directions of the room in the gas-phase) is occurring if their centres are in closer proximity than the sum of their radii [37,38,39]. Therefore, the CCS may be considered as the product of the squared sum of the radii and π. Accordingly, the mean calculated CCS translates into a mean projected ion radius of r_i_ = 5.6 Å (±0.5 Å SD) or a mean projected ion area A_i_ = 99 Å^2^ (±19 Å^2^ SD) considering the kinetic radius of molecular nitrogen [40]. Further, as the difference between momentum and collision cross section is up to 40% a converted area may be calculated as A_con_ = 63 Å^2^ (±13 Å^2^ SD).

Finally, the calculated radius r_i_, the calculated threshold ion radius for BBB^+^ compounds of r_i,max_ = 6.2 Å [41] and the converted ion radius r_i,con_ = 4.4 Å (±0.5 Å SD) may be compared with the dimensions present in the BBB. This reveals a key result of our study. In fact, the determined limit in molecular size fits the dimension of the pores (∅ = 10 Å [42]) that form temporarily during permeation of small molecules through a lipid-bilayer. It is assumed that the fatty acid alkyl chains forming the membrane kink due to molecular motion to allow permeation [42,43,44]. Further, the intercellular crevice of the BBB is sealed by tight junctions employing claudins, that were shown to express pore-like assemblies of 8–10 Å diameter [45], which perfectly matches the drug dimensions mentioned above. Consequently this allows the conclusion that small molecules may penetrate the brain also paracellulary if they are below the radius threshold [45]. In sum, it becomes evident that the BBB permeation is also determined by the size of a molecule. In other words, the BBB may be considered as a sieve and substances below a certain size limit (the dimension of the pores) are able to pass, if only passive diffusion is considered.

## 4. Conclusions

The collision cross section was proven to be a valuable additional descriptor of BBB permeation that provides spatial information using large data sets. A useful improvement over earlier approaches [13] is to consider the charge of a molecule at physiological pH to choose the ionic adduct (protonated or deprotonated ion) to mimic the biological scenario more accurately. This is possible with an in silico procedure. In the case of an instrumental determination of CCS the adduct is foremost depending on ionization parameters and molecular properties of investigated ions.

As mentioned above, the CCS is indeed mass dependent but exhibits more information than the molecular mass alone because it also reflects the molecular volume, shape and structure. Keeping in mind that these parameters influence the capability of a substance for BBB permeation makes CCS evaluation of pharmaceutical compounds attractive for prediction of cerebral pharmacokinetics. However, it is worth noting that we solely considered passive diffusion in this correlation of CCS with a drugs ability to penetrate the brain. Accordingly, a possible substrate character to a membrane transporter may fully change the corresponding cerebral pharmacokinetics. This however is also the case for other structure-derived physicochemical parameters that are commonly used.

Interestingly enough the computed molecular size aligns well with reported dimensions of pores that form temporarily upon membrane crossing of molecules. In addition, tight-junction claudins were reported to form pores of the same dimension. Accordingly, both an intra- and an intercellular passage of small-molecule drugs that are also predetermined by molecular size are plausible.

In conclusion, we emphasize the benefit of CCS for the drug development process alongside other descriptors, to optimize initial characterization of substances in terms of their ability to penetrate the blood-brain barrier. Considering the ease of either measurement or calculation of CCS with open access tools it is highly recommendable to include this parameter in preclinical testing to improve the validity of predicted cerebral pharmacokinetics.

## Figures and Tables

**Table 1:** Results of the scaffold diversity analysis according to DataWarrior output.

Data Set	Compounds (N)	Ring System with Atomic-No Subst. Pattern Scaffolds (Nrs)	Murcko Scaffolds (Nms)	Most Central Ring Scaffolds (Ncr)	Skeleton Scaffolds (Nsc)	Nrs/N	Nms/N	Ncr/N	Nsc/N
Adenot	1592	961	842	320	581	0.60	0.53	0.20	0.36
Adenot+	1282	683	672	276	447	0.53	0.52	0.22	0.35
Adenot−	310	215	168	68	150	0.69	0.54	0.22	0.48
Drugbank	3261	2101	1729	572	1107	0.64	0.53	0.18	0.34
Drugbank+	2841	1754	1462	478	903	0.62	0.51	0.17	0.32
Drugbank−	420	432	282	123	235	1.03	0.67	0.29	0.56
Li	400	324	222	118	164	0.81	0.56	0.30	0.41
Li+	267	222	144	87	105	0.83	0.54	0.33	0.39
Li-	133	149	88	54	78	1.12	0.66	0.41	0.59
Muehlbacher	362	233	148	86	117	0.64	0.41	0.24	0.32
Muehlbacher+	327	203	132	78	104	0.62	0.40	0.24	0.32
Muehlbacher−	35	46	25	21	22	1.31	0.71	0.60	0.63

**Table 2:** Ranges of relevant molecular descriptors of successful CNS drugs [8,9,13].

Descriptor	Range
Polar Surface Area	<60–70 Å
CCS	<200 Å^2^
log D 7.4	0–3
Molecular weight	<450 Da
log P	~2
No. H-Acceptors	<7
No. H-Donors	<3
No. Rotatable Bonds	<8
pKa	7.5–10.5 (neutral or basic, not acidic)

## Data Availability

All presented data are available as supplementary files online.

## Supplementary Materials

The following are available online at https://www.mdpi.com/article/10.3390/pharmaceutics13122141/s1, Table S1: manuscript-supplementary.xlsx and Jupyter File S2: BBB_DataAnalysis.ipynb.

## References

[1] Reichel A. (2009). Addressing Central Nervous System (CNS) Penetration in Drug Discovery: Basics and Implications of the Evolving New Concept. Chem. Biodivers..

[2] Pardridge W.M. (2007). Blood–brain barrier delivery. Drug Discov. Today.

[3] Reichel A. (2006). The Role of Blood-Brain Barrier Studies in the Pharmaceutical Industry. Curr. Drug Metab..

[4] Hammarlund-Udenaes M., Fridén M., Syvänen S., Gupta A. (2008). On the Rate and Extent of Drug Delivery to the Brain. Pharm. Res..

[5] Bauer H.-C., Krizbai I.A., Bauer H., Traweger A. (2014). “You Shall Not Pass”—Tight junctions of the blood brain barrier. Front. Neurosci..

[6] Abbott N.J. (2013). Blood–brain barrier structure and function and the challenges for CNS drug delivery. J. Inherit. Metab. Dis..

[7] Engelhardt B., Sorokin L. (2009). The blood–brain and the blood–cerebrospinal fluid barriers: Function and dysfunction. Semin. Immunopathol..

[8] Pajouhesh H., Lenz G.R. (2005). Medicinal chemical properties of successful central nervous system drugs. NeuroRX.

[9] Fong C.W. (2015). Permeability of the Blood–Brain Barrier: Molecular Mechanism of Transport of Drugs and Physiologically Important Compounds. J. Membr. Biol..

[10] Gerebtzoff G., Seelig A. (2006). In Silico Prediction of Blood−Brain Barrier Permeation Using the Calculated Molecular Cross-Sectional Area as Main Parameter. J. Chem. Inf. Model..

[11] Fischer H., Gottschlich R., Seelig A. (1998). Blood-Brain Barrier Permeation: Molecular Parameters Governing Passive Diffusion. J. Membr. Biol..

[12] Van Bree J.B., De Boer A.G., Danhof A., Ginsel L.A., Breimer D.D. (1988). Characterization of an “in vitro” blood-brain barrier: Effects of molecular size and lipophilicity on cerebrovascular endothelial transport rates of drugs. J. Pharmacol. Exp. Ther..

[13] Guntner A.S., Thalhamer B., Klampfl C., Buchberger W. (2019). Collision cross sections obtained with ion mobility mass spectrometry as new descriptor to predict blood-brain barrier permeation by drugs. Sci. Rep..

[14] D’Atri V., Causon T., Hernandez-Alba O., Mutabazi A., Veuthey J.L., Cianferani S., Guillarme D. (2018). Adding a new separation dimension to MS and LC-MS: What is the utility of ion mobility spectrometry?. J. Sep. Sci..

[15] Stow S.M., Causon T.J., Zheng X., Kurulugama R.T., Mairinger T., May J.C., Rennie E.E., Baker E.S., Smith R.D., McLean J.A. (2017). An Interlaboratory Evaluation of Drift Tube Ion Mobility–Mass Spectrometry Collision Cross Section Measurements. Anal. Chem..

[16] Siems W.F., Viehland L.A., Hill H.H. (2012). Improved Momentum-Transfer Theory for Ion Mobility. 1. Derivation of the Fundamental Equation. Anal. Chem..

[17] Mason E.A., McDaniel E.W. (1988). Transport Properties of Ions in Gases.

[18] Ross D.H., Xu L. (2021). Determination of drugs and drug metabolites by ion mobility-mass spectrometry: A review. Anal. Chim. Acta.

[19] Shrivastav V., Nahin M., Hogan C.J., Larriba-Andaluz C. (2017). Benchmark Comparison for a Multi-Processing Ion Mobility Calculator in the Free Molecular Regime. J. Am. Soc. Mass Spectrom..

[20] Zhou Z., Luo M., Chen X., Yin Y., Xiong X., Wang R., Zhu Z.-J. (2020). Ion mobility collision cross-section atlas for known and unknown metabolite annotation in untargeted metabolomics. Nat. Commun..

[21] Ross D.H., Cho J.H., Xu L. (2020). Breaking Down Structural Diversity for Comprehensive Prediction of Ion-Neutral Collision Cross Sections. Anal. Chem..

[22] Adenot M., Lahana R. (2004). Blood-Brain Barrier Permeation Models: Discriminating between Potential CNS and Non-CNS Drugs Including P-Glycoprotein Substrates. J. Chem. Inf. Comput. Sci..

[23] Zhao Y.H., Abraham M.H., Ibrahim A., Fish P.V., Cole S., Lewis M.L., de Groot M.J., Reynolds D.P. (2007). Predicting Penetration Across the Blood-Brain Barrier from Simple Descriptors and Fragmentation Schemes. J. Chem. Inf. Model..

[24] Wishart D.S., Knox C., Guo A.C., Shrivastava S., Hassanali M., Stothard P., Chang Z., Woolsey J. (2006). DrugBank: A comprehensive resource for in silico drug discovery and exploration. Nucleic Acids Res..

[25] Li H., Yap C.W., Ung C.Y., Xue Y., Cao Z.W., Chen Y.Z. (2005). Effect of Selection of Molecular Descriptors on the Prediction of Blood−Brain Barrier Penetrating and Nonpenetrating Agents by Statistical Learning Methods. J. Chem. Inf. Model..

[26] Daina A., Zoete V. (2016). A BOILED-Egg to Predict Gastrointestinal Absorption and Brain Penetration of Small Molecules. ChemMedChem.

[27] Daina A., Michielin O., Zoete V. (2017). SwissADME: A free web tool to evaluate pharmacokinetics, drug-likeness and medicinal chemistry friendliness of small molecules. Sci. Rep..

[28] Muehlbacher M., Spitzer G.M., Liedl K.R., Kornhuber J. (2011). Qualitative prediction of blood–brain barrier permeability on a large and refined dataset. J. Comput. Aided Mol. Des..

[29] Cheng F., Li W., Zhou Y., Shen J., Wu Z., Liu G., Lee P.W., Tang Y. (2012). admetSAR: A Comprehensive Source and Free Tool for Assessment of Chemical ADMET Properties. J. Chem. Inf. Model..

[30] Chong J., Wishart D.S., Xia J. (2019). Using MetaboAnalyst 4.0 for Comprehensive and Integrative Metabolomics Data Analysis. Curr. Protoc. Bioinform..

[31] Breiman L. (2001). Random Forests. Mach. Learn..

[32] Chhabra S., Kumar S., Parkesh R. (2021). Chemical Space Exploration of DprE1 Inhibitors Using Chemoinformatics and Artificial Intelligence. ACS Omega.

[33] Langdon S.R., Brown N., Blagg J. (2011). Scaffold Diversity of Exemplified Medicinal Chemistry Space. J. Chem. Inf. Model..

[34] Minikel E.V. Properties of CNS Drugs vs. All FDA-Approved Drugs. https://www.cureffi.org/2013/10/04/properties-of-cns-drugs-vs-all-fda-approved-drugs/.

[35] Roy D., Hinge V.K., Kovalenko A. (2019). To Pass or not to Pass: Predicting the Blood–Brain Barrier Permeability with the 3D-RISM-KH Molecular Solvation Theory. ACS Omega.

[36] Gabelica V., Shvartsburg A.A., Afonso C., Barran P., Benesch J.L.P., Bleiholder C., Bowers M.T., Bilbao A., Bush M.F., Campbell J.L. (2019). Recommendations for reporting ion mobility Mass Spectrometry measurements. Mass Spectrom. Rev..

[37] Wyttenbach T., Bleiholder C., Bowers M.T. (2013). Factors Contributing to the Collision Cross Section of Polyatomic Ions in the Kilodalton to Gigadalton Range: Application to Ion Mobility Measurements. Anal. Chem..

[38] Feng L., Dunaway K. Collisional Cross Section. https://chem.libretexts.org/@go/page/1403.

[39] Hinnenkamp V., Klein J., Meckelmann S.W., Balsaa P., Schmidt T.C., Schmitz O.J. (2018). Comparison of CCS Values Determined by Traveling Wave Ion Mobility Mass Spectrometry and Drift Tube Ion Mobility Mass Spectrometry. Anal. Chem..

[40] Aguilar-Armenta G., Patiño-Iglesias M.E., Leyva-Ramos R. (2003). Adsorption Kinetic Behaviour of Pure CO2, N2 and CH4 in Natural Clinoptilolite at Different Temperatures. Adsorpt. Sci. Technol..

[41] Guntner A.S. (2020). Into the Depth: The Key Role of Modern Analytical Chemistry in Pharmaceutical Development and Medicinal Research. Ph.D. Thesis.

[42] Marrink S.J., Jähnig F., Berendsen H.J. (1996). Proton transport across transient single-file water pores in a lipid membrane studied by molecular dynamics simulations. Biophys. J..

[43] Träuble H. (1971). The movement of molecules across lipid membranes: A molecular theory. J. Membr. Biol..

[44] Kadry H., Noorani B., Cucullo L. (2020). A blood–brain barrier overview on structure, function, impairment, and biomarkers of integrity. Fluids Barriers CNS.

[45] Irudayanathan F.J., Wang N., Wang X., Nangia S. (2017). Architecture of the paracellular channels formed by claudins of the blood–brain barrier tight junctions. Ann. N. Y. Acad. Sci..

